# Improving cellular phylogenies through the integrated use of mutation order and optimality principles

**DOI:** 10.1016/j.csbj.2023.07.018

**Published:** 2023-08-02

**Authors:** Sayaka Miura, Tenzin Dolker, Maxwell Sanderford, Sudhir Kumar

**Affiliations:** a Institute for Genomics and Evolutionary Medicine, Temple University, Philadelphia, PA 19122, USA; b Department of Biology, Temple University, Philadelphia, PA 19122, USA

**Keywords:** Single-cell sequencing, Phylogeny, Mutational history, Tumor evolution, Metastasis

## Abstract

The study of tumor evolution is being revolutionalized by single-cell sequencing technologies that survey the somatic variation of cancer cells. In these endeavors, reliable inference of the evolutionary relationship of single cells is a key step. However, single-cell sequences contain many errors and missing bases, which necessitate advancing standard molecular phylogenetics approaches for applications in analyzing these datasets. We have developed a computational approach that integratively applies standard phylogenetic optimality principles and patterns of co-occurrence of sequence variations to produce more expansive and accurate cellular phylogenies from single-cell sequence datasets. We found the new approach to also perform well for CRISPR/Cas9 genome editing datasets, suggesting that it can be useful for various applications. We apply the new approach to some empirical datasets to showcase its use for reconstructing recurrent mutations and mutational reversals as well as for phylodynamics analysis to infer metastatic cell migrations between tumors.

## Graphical Abstract

**Figure:**
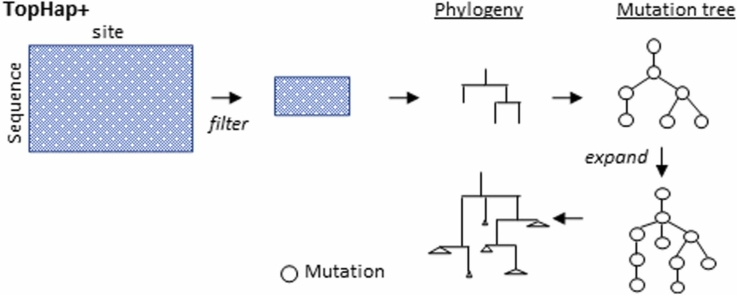

## 1 Introduction

In cancer, somatic mutations occur continuously and are subjected to natural selection, resulting in the ongoing evolution of tumor cell populations within a patient [1], [2], [3], [4]. Genomic surveys of tumor cell populations have enhanced our understanding of the patterns of adaptive mutations, dynamics of mutational processes, metastatic cell migrations, and patterns of gene expression changes [4], [5], [6], [7], [8], [9], [10], [25]. Such studies will become more commonplace with rapid advances in single-cell sequencing technologies that readily reveal genetic variation among individual tumor cells for studying tumor evolution at a cellular resolution [11], [12], [13].

However, the analysis of single-cell sequence datasets can be challenging owing to the presence of missing data as well as false-positive and false-negative detection of mutations [12], [13], [14]. Doublet sequences, where more than one cell is accidentally sequenced together, also contribute to sequencing errors and complicate analyses [12], [14]. Consequently, computational methods have been developed to improve the quality of single-cell sequences and impute missing bases, e.g., BEAM [13]. Also, methods that allow for the presence of sequencing errors have been developed, affording a more accurate inference of cellular phylogenies and tumor dynamics [13], [14], [15], [16], [17], [18].

**Fig. 1:**
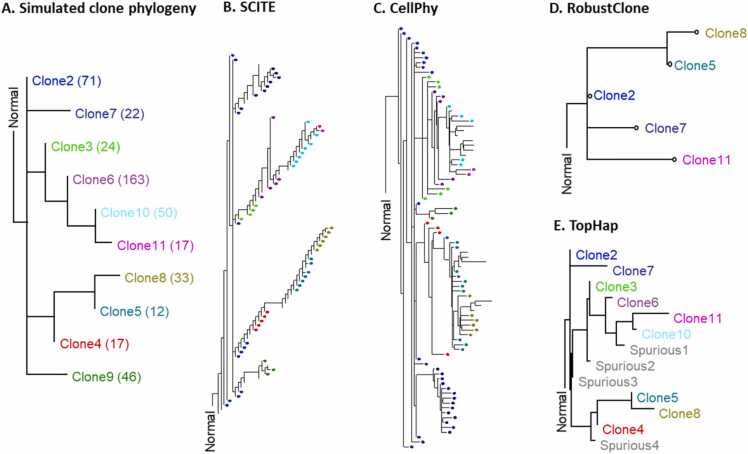
**Simulated (A) and inferred clone phylogeny using SCITE (B), CellPhy (C), RobustClone (D), and TopHap (E).** (**A**) A simulated phylogeny of 10 clones with 100 single nucleotide variants (SNVs). The number of cells sampled for each clone is shown in parentheses. Cell sequences were generated by computer simulations along the clone phylogeny, with sequencing errors and missing bases introduced in the resulting alignment. The doublet sequencing rate, false positive rate, false negative rate, and rate of missing data were set to 10%, 1%, 20%, and 20%, respectively. (**B-E**) Clones and phylogenies inferred by using (**B**) SCITE, (**C**) CellPhy, (**D**) RobustClone, and (**E**) TopHap methods. BEAM was applied prior to TopHap in order to deal with missing data and sequencing errors, whereas other methods intrinsically model these errors. Phylogenies were rooted using the normal cell sequence. For SCITE and CellPhy, inferred clones are colored based on the simulated clones, as a large number of clones were produced. Tips without color are clones involved in doublet sequencing in the simulated datasets.

Although many available computational methods model sequencing errors to infer mutation order and phylogeny of cells or clones [13], [14], [15], [16], [17], [18], there is still a need for methods to further improve inference. We motivate this need by applying available methods to infer a cellular phylogeny using a synthetic dataset for which the true phylogeny is known (Fig. 1**A**). For this dataset, a sophisticated mutation ordering method, SCITE [16], produced a clone phylogeny with many spurious clones, which resulted in a much larger phylogeny than that simulated (Fig. 1**B**). CellPhy, which uses a phylogenetic optimality principle of Maximum Likelihood [18], incorrectly predicted that almost all the cell sequences were distinct (Fig. 1**C**). In contrast, an approach to cluster cells by the similarity of observed cell sequences (RobustClone [17]) greatly underestimated the number of distinct clones (Fig. 1**D**). Therefore, the presence of sequencing errors adversely impacted different methods in different ways, despite the fact that some of these methods specifically model sequencing errors during clone and phylogeny inference.

Previously, we developed a method to distinguish phylogenetic signals from sequencing errors by restricting the analysis to haplotypes containing high-frequency variants [19]. This method (called TopHap) performed better than others (Fig. 1**E**), but still produced a phylogeny with a few spurious clones (false positives). Also, it contained one missing clone (false negative), which occurred with a low frequency (<50 cells per clone). Based on these examples, it is clear that some current methods do not perform well on some datasets, and there is a room to improve these methods. In this study, we explore one such possibility in which we sought to advance the TopHap method by integrating it with mutation ordering analysis to detect spurious clones and identify additional clones (rare clones containing lower frequency variants) as well as their placement in the cell phylogeny. We refer to the new approach as TopHap+ , whose reliability and usefulness were evaluated in this article. In the following, we describe TopHap+ and then investigate its performance using simulated and empirical datasets.

## 2 Materials and methods

### 2.1 TopHap+ approach

**Fig. 2:**
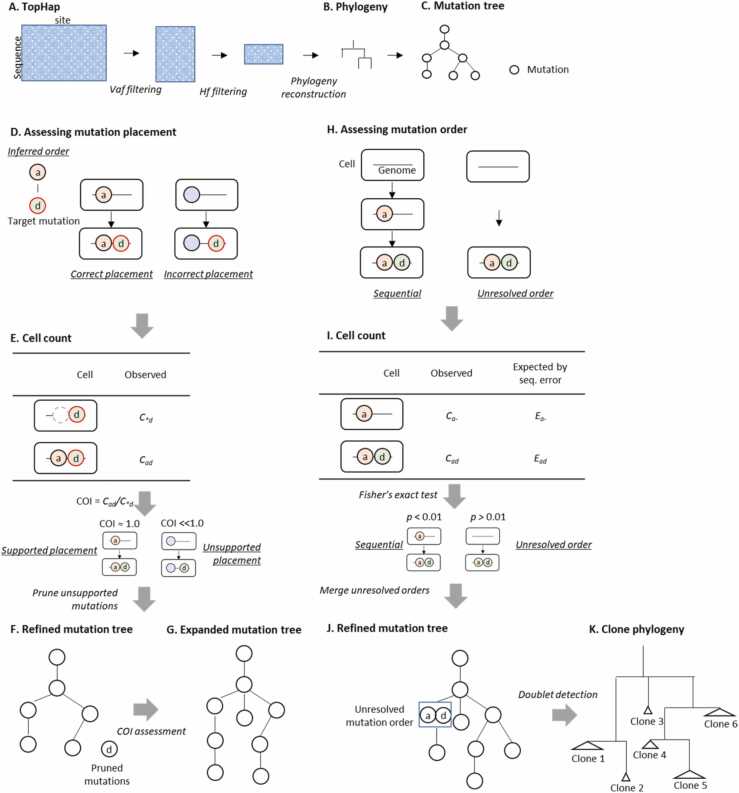
**An overview of TopHap+ .** (**A**) Genomic sites with mutation frequencies lower than the variant allele frequency (VAF) threshold are removed from the full alignment. Then, haplotype sequences with lower than haplotype frequencies (HF) are removed. (**B**) A phylogeny of haplotypes is inferred, and the induced mutation tree is produced (**C**). (**D** and **E**) Mutation placement is assessed by using COI, and (**F**) spuriously placed mutations are pruned. (**G**) Mutations that are not included in the mutation tree are considered for reattachment using COI. (**H** and **I**) Inferred mutation order is assessed by counting the number of cells with target mutations, and (**J**) the mutation tree is refined. (**H**) Doublet cells are identified and pruned, and a final clone phylogeny is produced.

The TopHap+ uses an alignment of cell sequences (Fig. 2**A**). The first step is to refine cell sequences using an available method, such as BEAM [13], in order to obviate the need for a model to deal with false positive and false negative mutation calls, such as those required by SCITE. Following the TopHap approach [19], we first select high-frequency SNVs, at a desired variant allele frequency (VAF) threshold (default: >5 cells), to construct an alignment of haplotypes. High-frequency haplotypes, which correspond to major tumor clones, are then selected at a desired haplotype frequency (HF) threshold (default: >5 cells), and a phylogeny of major clones is inferred (Fig. 2**B**).

Next, we perform mutation ordering analysis to assess the phylogeny inferred above. To do so, the phylogeny is converted into a mutation tree by reconstructing ancestral sequences at internal nodes of the phylogeny using the Maximum Parsimony (MP) method (Fig. 2**C**) [20]. The most likely ancestral state is assigned to every internal node of the tree at each site. A mutation is said to have occurred when there is a difference in bases between the ancestral and descendant nodes. If the placement of a given mutation (say *d*) in the mutation tree is reliable (i.e., the phylogeny is correct), cells that carry *d* should also carry the mutation (say *a*) that occurred in the immediately ancestral branch (Fig. 2**D**). TopHap+ calculates the ratio of the proportions of cells with both mutations (*C*_*ad*_) and with the descendant mutation (C_*d_), which is referred to as the co-occurrence index (COI [21]) (Fig. 2**E**),

(1) COI = C_ad_/C_*d_·

COI is calculated for all inferred pairs of descendant (*d*) and ancestor (*a*) mutations. COI is calculated using the originally observed cell sequences before BEAM refinement, which avoids any bias in the imputed bases and error corrections. When multiple mutations are mapped to the same branch, the average COI for all the mutations is used. TopHap+ prunes tips and subtrees if the COI is too low (we used a cut-off of 0.3) (Fig. 2**F**). Note that tips and mutations removed in this way are reconsidered for attachment in the clone phylogeny, as outlined below.

Next, TopHap+ inspects the alignment and finds all the mutations that are missing from the current mutation tree, including those with frequencies lower than the previous cut-off (Fig. 2**G**). TopHap+ calculates the COI of a candidate variant with each of the other mutations, and the candidate mutation is attached to the mutation tree if its COI is greater than the desired threshold (we used a cut-off of 0.4). Search for the best place to attach the candidate variant starts from the tip of the mutation tree, and the deepest attachment position is selected. TopHap+ begins with candidate mutations with the highest VAF, and the mutation tree is expanded continuously.

At the end, TopHap+ derives candidate clone sequences by accumulating mutations from the root to each node of the mutation tree. All the candidate sequences are compared with the cell sequences in the original alignment to assign cells to clones, based on the highest sequence similarity. TopHap+ estimates the false-positive detection rate of mutations (FPR) and false-negative rate (FNR) by comparing observed cell sequences with their matched clone sequences. The FNR is the proportion of germline (wild-type) bases that are observed at the positions of predicted mutant bases, while FPR is the proportion of mutant bases that are observed at the positions of predicted wild-type bases.

Then, we re-test the relationship of mutations on adjacent branches by using the FNR to detect if any intermediate clone is spuriously inferred in mutation ordering analysis due to sequencing errors resulting in incorrectly observing germline (wild-type) bases (e.g., Fig. 1**B**). When mutations occur sequentially, the data should contain real intermediate cells that carry only the early (ancestor) mutation without the later (descendant) mutation (Fig. 2**H**). Thus, TopHap+ tests if the number of predicted intermediate cells with only early mutation and lacking the later mutation (*C*_*a-*_) is significantly larger than the expected number of spurious intermediate cell sequences due to false-negatives of the descendant mutation (*E*_*a-*_). Given the FNR and the total number of cells with the early mutation (*C*_*a-*_ + *C*_*ad*_), *E*_*a-*_ is calculated by,

(2) *E*_*a-*_ = (*C*_*a-*_ + *C*_*ad*_) × FNR,

where *C*_*ad*_ is the number of observed cells that carry both earlier and later mutations. The expected number of the descendant cells with both early and later mutations is then calculated as,

(3) *E*_*ad*_ = (*C*_*a-*_ + *C*_*ad*_) – *E*_*a-*_·

TopHap+ conducts Fisher's exact test using *C*_*a-*_, *C*_*ad*_, *E*_*a-*_, and *E*_*ad*_ (Fig. 2**I**), with a significant *p*-value (< 0.01) supporting the sequential occurrence of the two mutations. Since we did not observe elevated false positive detection of clones (Fig. 4**A**), there was no need to make any adjustments for multiple-testing. TopHap+ performs this test for each pair of ancestor-descendant mutations in a given mutation tree and accordingly refines the mutation tree (Fig. 2**J**) by removing likely spurious ancestral clones.

To convert the mutation tree into a final clone phylogeny, TopHap+ again assigns a clone sequence to each cell, i.e., the clone annotation (see above). TopHap+ also tests if a cell sequence is that of a doublet cell, i.e., more than one cell has been sequenced together. Since a doublet cell carries mutations from more than one cell, each observed cell sequence is additionally paired with the most similar expected sequence that carries the largest number of observed mutations not found in the assigned clone sequence. The expected doublet cell sequence is then constructed by adding all mutations from the two annotated expected sequences. Following a previous study [16], TopHap+ calculates the likelihood of observing a sequence (D) with *n* SNVs given an expected sequence (T), FNR, and FPR,

(4) $$L(\mathrm{D|T},\mathrm{FNR},\mathrm{FPR})=\Pi_{i=1}^{n}P(D_{i}|T_{i}),$$

where.

P(D_*i*_ = wild-type | T_*i*_ = wild-type) = 1 − FPR,

P(D_*i*_ = mutant | T_*i*_ = wild-type) = FPR,

P(D_*i*_ = wild-type | T_*i*_ =mutant) = FNR,

(5) P(D_*i*_ = mutant | T_*i*_ = mutant) = 1 − FNR·

The D_*i*_ and T_*i*_ represent the *i*-th SNV in observed and expected sequences, respectively. TopHap+ calculates the log-likelihood (*ln*L) by giving each of the expected singleton and doublet sequences and performs the likelihood ratio test, i.e.,

(6) LR = 2(lnL(D | T = doublet) – lnL(D | T = singleton)) with *df* = 1·

If the doublet fits better (*p* < 0.01), then the cell is annotated as a doublet and is removed from the clone annotation (Fig. 2**K**). A detailed description of TopHap+ can be found in the Supplementary Note.

### 2.2 Assembly and analysis of simulated datasets

We obtained 110 simulated datasets with 100 – 2000 cells from a previous study [17]. For all simulated datasets, the doublet rate, FPR, FNR, and rate of missing data were 10%, 1%, 20%, and 20%, respectively. The number of tumor clones was 5 – 50. We randomly selected 100 SNVs when a simulated dataset contained > 100 variants because < 100 mutations are often profiled through targeted single-cell sequencing, e.g., [22], [23]. These datasets were generated under the assumption of the infinite sites model. The topology of each clone phylogeny was distinct and was generated randomly [17].

TopHap+ analysis was performed with the default VAF and HF thresholds (5 cells). We compared the performance of TopHap+ with SCITE, CellPhy, and RobustClone, which have been reported to perform well in previous studies [13], [17], [18]. The SCITE [16] analysis was performed with 900,000 MCMC chains, and the number of repetitions was set to one. The simulated FPR and FNR were provided, and the option to attach cells to an inferred mutation tree was selected. Clones were defined as groups of cells attached at the same node of the mutation tree.

Following the default setting, RobustClone [17] was performed by the robust principal component analysis (RPCA) to refine observed cell genotypes, followed by clustering cells, clone annotation, and clone genotype prediction. A clone phylogeny was inferred using the maximum likelihood method with the Jukes-Cantor substitution model in MEGA-CC [24]. The phylogeny was rooted using the normal cell sequence, which had no mutations.

CellPhy [18] analysis was performed with the GTR substitution model for diploid genotypes, where the stationary frequencies were obtained from the ML estimate (GTGTR4 +FO+E option). A normal cell, which did not contain any mutations, was set to be the outgroup. Mutations were then mapped on the inferred phylogeny using the “mutmap” option. The same substitution model was selected, and the branch length optimization was disabled. Similarly, a normal cell was assigned to be the outgroup. Predicted cell sequences were generated by accumulating mutations from the normal cell to a tip of the phylogeny, excluding mutations that were mapped at the external branches because inferred cell phylogenies had spuriously very long external branches (e.g., Fig. 1**C**). Clones were defined as groups of cells with identical inferred sequences.

### 2.3 Evaluating the accuracy of inferred clones and phylogenies

To evaluate the accuracy of inferred clone phylogenies, we computed the RF distance using the phangorn R package [26]. RF distance is a standard approach to evaluate the similarity of two phylogenies in molecular evolution and phylogenetics. We converted simulated cell phylogenies to clone phylogenies, where clones were groups of cells with no sequence differences. We paired each simulated (true) clone to an inferred clone that contained the largest number of cells that belonged to the simulated clone. We allowed a single inferred clone to be paired with more than one simulated clone. When more than one simulated clone was paired, the tip of the inferred phylogeny was duplicated because the RF computation required the same number of tips in the phylogenies. Also, we pruned inferred and true clones that were not paired. For example, a simulated clone did not have an inferred clone pair when those simulated cells were excluded in the inferred clone annotation, e.g., false-positive detection of doublets. We counted the number of total partitions and those not found in the other phylogeny for each phylogeny. The number of pruned simulated clones for the RF computation was counted as the number of partitions not found in the inferred phylogeny. Overall, RF is not designed to be used for phylogenies with different numbers of tips, and errors of missing and additional clones cannot be accurately assessed using RF distances. Therefore, to further assess errors in the size of inferred phylogeny (the number of tips), we also reported the difference in the number of clones inferred and the true clone count.

### 2.4 Assembly and analysis of empirical datasets

We obtained targeted single-cell sequencing data of metastatic ovarian cancers (Patient 3 and Patient 9 datasets), where high-frequency SNVs were first identified in the bulk-tumor sequencing data, and then targeted in the single-cell sequencing [27]. The number of cells was 672 and 420, and the number of SNVs was 84 and 43, for Patient 3 and Patient 9, respectively. In the TopHap+ analysis, we used the 5% VAF and 10 cells HF thresholds, because the use of more relaxed default thresholds (5 cells) seemed to retain too many spurious haplotypes hampering the phylogenetic method, i.e., more than half of inferred clones were removed by the mutation ordering analysis due to spurious mutation placements (Supplementary Fig. S3).

We also obtained four datasets with > 1000 cells containing metastatic tumor cells that were generated through lineage tracing technology using CRISPR/Cas9 genome editing (dataset IDs: 3724, 3508, 3515, and 3454) [28]. For each dataset, cells were engineered with recording “target sites,” and heritable indels were accumulated over time, which were subsequently sequenced. We transformed the indel matrix into an alignment of binary variants corresponding to the presence and absence of each indel. Since sequencing errors rarely happen in the lineage tracing technology, we did not refine observed cell sequences at the first step of TopHap+ . In the TopHap+ analysis, VAF and HF thresholds were set to 1%, and mutations that were shared by > 30 (3724 and 3508), > 20 (3515), and > 15 cells (3454) were subject to the mutation ordering analysis to avoid a long computation time.

### 2.5 Using PathFinder in conjunction with TopHap+ in empirical data analysis

Inferred TopHap+ phylogeny can be used for downstream analysis. As an example, we illustrated the inference of metastasis cell migration events from TopHap+ phylogeny using PathFinder [29]. Inferred clone sequences and tumor sites that contain clones by TopHap+ were provided. When more than one section of a tumor site was sampled, we merged these sections into a single tumor site because our interest was to infer migration paths between tumor sites. We assumed a clone was present in a tumor site when at least one cell was detected. PathFinder analysis was performed by providing the primary tumor site information. For a polytomy, at most 100 different tumor membership states were examined. When no tumor site was predicted with > 0.15 posterior probability at a given ancestral node, the site with the highest probability was selected.

## 3 Results

### 3.1 Accuracy of TopHap+ clone phylogeny

**Fig. 3:**
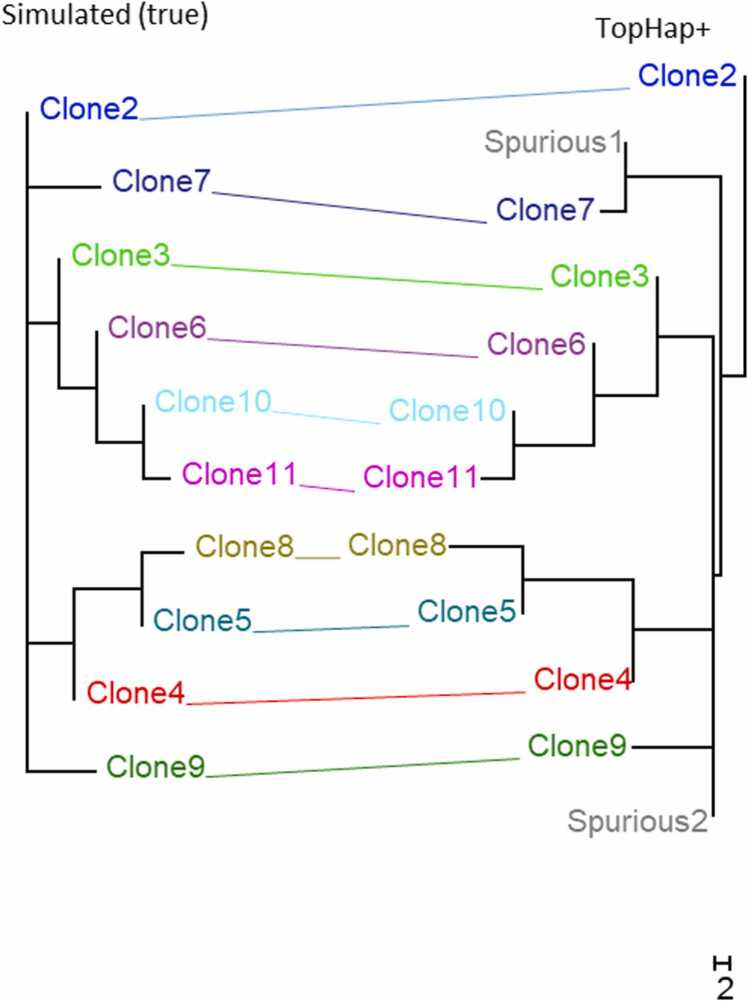
**Inferred phylogeny using TopHap+ .** Sequence alignment simulated using the phylogeny in Fig. 1 (on the left) was used. TopHap+ clones and their phylogeny is compared with the true phylogeny. TopHap+ phylogeny contains two spurious clones (gray color), and thus, the number of tips is larger than the simulated phylogeny.

We first tested the absolute performance of TopHap+ and then compared it with the other methods for the example dataset presented in Fig. 1**A**. TopHap+ clone phylogeny agreed well with the simulated phylogeny (Fig. 3), which was better than the performance of other methods (Fig. 1). For this dataset, the mutation ordering analysis in TopHap+ removed many spurious clones produced by TopHap. Also, it identified the missing clone that occurred with a low frequency (Fig. 1**E**). Unlike SCITE, TopHap+ did not produce many incorrect intermediate clones (Fig. 1**B**). Also, CellPhy predicted all cell sequences to be distinct, which was not the case for TopHap+ (Fig. 1**C**). TopHap+ also did not underestimate the clone count, a problem seen for RobustClone (Fig. 1**D**). Therefore, TopHap+ performed well on this example dataset, which we had selected for illustrating likely improvements offered by TopHap+ .

To obtain a more general assessment of the improvements offered by TopHap+ , we next analyzed additional simulated datasets available from an independent study [17]. There were a total of 110 datasets, each with 100 variants, the number of cells from 100 to 2000, and the number of clones from 5 to 50. We used simulated datasets because the ground truth about cell phylogeny is known. By the way, the cell phylogenies used to simulate these 110 datasets were different from each other.

**Fig. 4:**
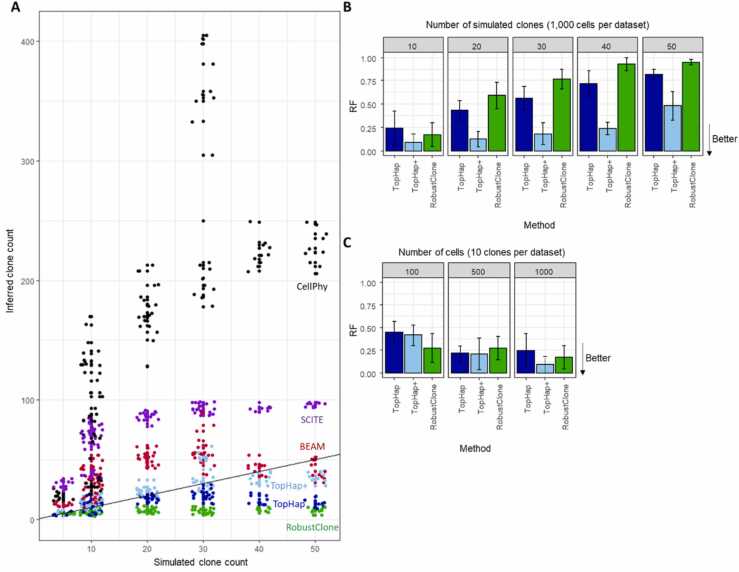
**The performance of TopHap+ using simulated datasets.** (**A**) Simulated and inferred clone counts. Each dot represents a dataset. The black line is the one-to-one line. A plot that contains < 100 inferred clones is shown in Supplementary Fig. S4. (**B** and **C**) Accuracy of inferred clone phylogeny for datasets with an increasingly larger number of clones simulated (**B**) and cells (**C**). In panel **B**, datasets contained 1000 cells, so the number of cells per clone decreased with the increasing number of clones. In panel **C**, there were 10 clones in each dataset, so the number of cells per clone increased as the increasing number of cells. RF was computed between inferred and simulated clone phylogenies for each method. The average RF across the datasets is plotted, and an error bar represents the standard deviation. We included only datasets with at least three inferred clones. CellPhy and SCITE were not included because the number of inferred clones was extensively overestimated.

The number of clones produced by TopHap+ was the most similar to the actual number of clones simulated, as compared to other methods (Fig. 4**A**). When the number of simulated clones was large, i.e., 50, BEAM performed better than TopHap+ . However, BEAM produced too many clones for many datasets. While the simple filtering of low-frequency sequences (TopHap, the first step in TopHap+) resulted in distinct sequences similar in the count to the number of simulated clones when they were fewer than 20, such filtering generally underestimated the number of clones for datasets containing many simulated clones.

Also, the inferred clone phylogeny by TopHap+ was more accurate than TopHap, as the RF distance was smaller (Figs. 4**B** and 4**C**). These clone phylogenies were more accurate for datasets with fewer clones (Fig. 4**B**) because TopHap+ predicted fewer clones for datasets with many clones. Note that the variation of inference accuracies within the same number of simulated clones is a function of different simulation conditions, e.g., the topology of clone phylogeny. Similarly, TopHap inferred phylogenies more accurately when the number of simulated clones in a dataset was smaller, but TopHap+ clone phylogenies were always better.

Also, TopHap+ phylogeny was particularly more accurate when the number of cells in the dataset was larger (Fig. 4**C**). On the other hand, TopHap’s accuracy was better for datasets with 500 cells than 100 cells, while its accuracy did not become better for larger datasets, i.e., 1000 cells. Since the mutation ordering analysis in TopHap+ uses the pattern of co-occurring mutations, datasets with a larger number of cells were expected to show a clearer pattern of mutations among cells resulting in better accuracies.

We lastly compared the performance of TopHap+ with other methods. As observed in the example data analysis (Fig. 1), SCITE and CellPhy generally produced too many clones, while RobustClone produced too few (Fig. 4**A**). TopHap+ produced more accurate phylogenies than RobustClone for datasets with a large number of clones (≥20 clones) and a large number of cells (Figs. 4**B** and 4**C**). Note that since RobustClone performed slightly better than TopHap+ for datasets with a smaller number of cells (100), RobustClone may be selected for small datasets. We could not evaluate the accuracy of inferred clone phylogeny by CellPhy and SCITE, because they produced too many clones, i.e., the inferred phylogenies do not look similar to the simulated phylogenies. Overall, TopHap+ performed similarly to or better than the other methods.

### 3.2 Reconstruction of metastatic cell migration events using TopHap+

Next, we present two applications of TopHap+ in empirical data analysis and demonstrate the usefulness of TopHap+ in actual empirical data analysis. We first show an analysis of metastatic cell migration events using two targeted single-cell sequencing data of metastatic ovarian cancers, i.e., Patient 9 and Patient 3 datasets [27].

**Fig. 5:**
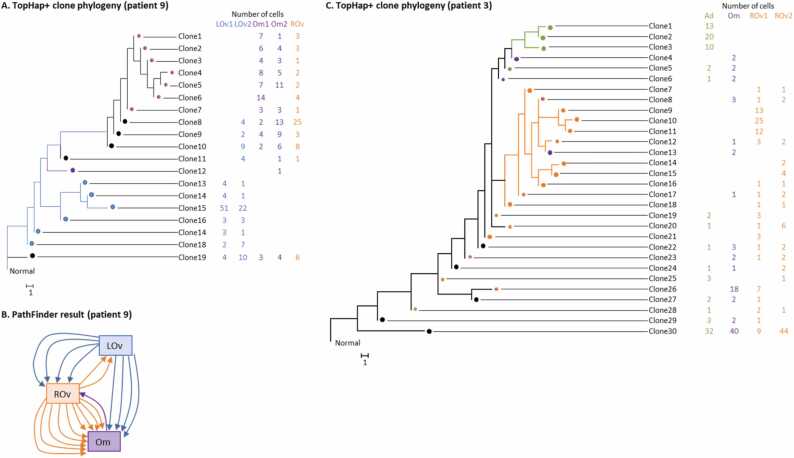
**Inferred clone phylogeny and metastasis migration paths using ovarian cancer data.** The Patient 9 and Patient 3 datasets [27] were analyzed using TopHap+ . (**A**) Inferred clone phylogeny (Patient 9). Cells were sampled from the left ovary (LOv1 and LOv2), right ovary (ROv), and omentum (Om1 and Om2). The number in a table is the number of cells. A circle at a tip of a phylogeny is a clone, and the color indicates tumor sites that contain a clone. A black circle represents that all tumors contain a clone. (**B**) Inferred metastatic cell migration history using PathFinder. Each arrow indicates a migration path. (**C**) Inferred clone phylogeny (Patient 3). Cells were sampled from the right ovary (ROv), omentum (Om), and adnexa (Ad). PathFinder did not complete the computation within a day, so the result was not shown.

Patient 9′s data consisted of 420 cells and 43 SNVs. The cells were obtained from the left ovary (two sections), right ovary (one section), and omentum (two sections). Fig. 5**A** shows the TopHap+ clone phylogeny. The number of cells annotated for each clone is shown at the tip of the phylogeny. As observed in the simulation study, the number of TopHap+ clones was larger than TopHap (19 and 6, respectively; Supplementary Fig. S5A).

The inferred phylogeny is ladder-like, suggesting that this tumor evolved under the linear cancer progression model [7]. All early clones were found within the left ovary, which was, thus, potentially the site where the tumor was initiated. Since the original article did not specify the location of the primary tumor, we assumed that the left ovary was the primary tumor to reconstruct the history of metastatic cell migration events. Then we inferred the metastatic cell migration history using PathFinder [29], which predicted that the right ovary and omentum were seeded by the left ovary through multiple cell migration events, i.e., there were multi-clone seeding events (Fig. 5**B**). In addition, PathFinder also predicted multi-clone seeding events from the right ovary to omentum. These seeding events explain why many clones are shared by tumors, which was also suggested by [7]. However, TopHap+ identified many more clones than [7], offering a more detailed result.

We next analyzed the Patient 3 dataset (84 SNVs with 672 cells), where cells were sampled from the right ovary (two samples), omentum, and adnexa. TopHap+ identified 31 tumor clones (Fig. 5**C**) compared to six clones deduced in the original study. Since the original study identified the clones through a cell clustering approach, the number of clones should have been underestimated, as we observed in our simulation study (i.e., RobustClone, which is based on a clustering approach). Nevertheless, the basic structure of TopHap+ phylogeny was similar to that reported in the original study, e.g., the early clones were found in all the tumor sites in both inferences from the TopHap+ and the original study, validating the TopHap+ inference. Also, as observed in our simulation study, the number of TopHap+ clones was larger than TopHap (8; Supplementary Fig. S5B).

In contrast to the Patient 9 dataset, in the TopHap+ inference, early clones were found in all the tumor sites. This observed pattern of clone sharing between tumors complicated a reliable reconstruction of metastatic migration history, i.e., PathFinder failed to produce a cell migration history. Nevertheless, late-arising clones fell into two major clades, one found only in the omentum and adnexa, while the other clade was composed of clones from the omentum and ovary. This pattern suggested that clones migrated between the omentum and adnexa and between the omentum and ovary at a later time. Since many clones were shared by more than one tumor, multi-clone seeding events should have frequently happened for this patient as well.

### 3.3 Detection of recurrent mutations and losses of mutations using TopHap+

A previous study analyzed the Patient 3 and 9 datasets using SCITE, which assumes that the same mutation does not occur multiple times at the same genomic position [15]. It reported that the violation of this assumption might result in mutation trees that looked different from when recurrent mutations and losses of mutations are allowed, i.e., recurrent mutations and losses of mutations were inferred by comparing the fit between with and without the additional model of recurrent and reversing mutation events in mutation ordering analysis [15].

**Fig. 6:**
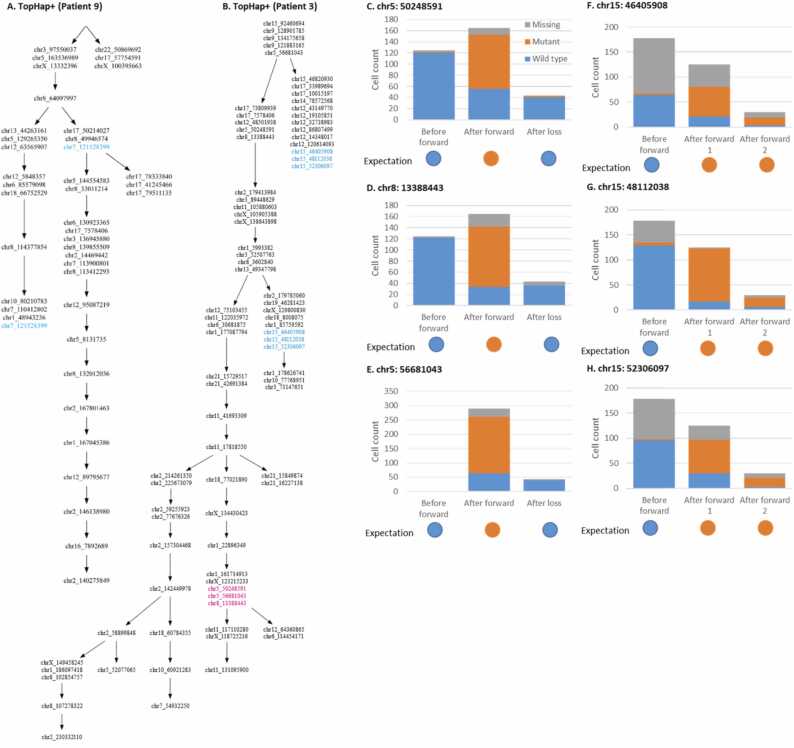
**Inferred recurrent mutations and losses of mutations.** (**A** and **B**) Inferred mutational histories by TopHap+ for Patient 9 (**A**) and Patient 3 (**B**). Inferred recurrent mutations and losses of mutations are shown with blue and red letters, respectively. (**C-H**) The number of bases observed among cells at the genomic positions of the predicted recurrent mutations and losses of the mutations by TopHap+ (Patient 3).

Therefore, we next compare TopHap+ inferences, where an additional model of recurrent and reversing mutation events is not required, with those from the previous study [15]. For the Patient 9 dataset, TopHap+ ordered 41 mutations and predicted the recurrent mutation (chr7: 121,528,399) that was identified in the previous study (Fig. 6**A**). Similarly, for the Patient 3 dataset, TopHap+ identified the loss of a mutation (chr5: 50,248,591) that was reported in the previous study (Fig. 6**B**). Also, both of the inferred mutation trees by TopHap+ were similar to those from the previous study.

Interestingly, TopHap+ additionally identified three recurrent mutations and two more losses of mutations for Patient 3, which were not found in the previous study (Fig. 6**B**). Since the inferred mutation order agreed well with the previous study, these detections were unlikely affected by errors in the inference. We also examined these mutations and found that observed bases supported these TopHap+ inferences (Fig. 6**C-6H**). For example, most cells that were affected by the inferred loss of the mutation, chr5: 50,248,591, did not have this mutation, while this mutation was observed among the cells that evolved after the forward mutation but that were not affected by the loss of the mutation, validating the inference of TopHap+ (Fig. 6**C**). In the case of inferred recurrent mutations, cells that were predicted to be affected by the recurrent mutations actually carried the mutant base, while those not affected did not, which similarly supported the TopHap+ inferences (Fig. 6**F-6H**). However, we noticed that the number of cells affected by the backward mutations was small. Since the false-negative detection rate of mutations is very high in single-cell sequencing data, inferred loss of mutation can be potentially spurious, i.e., predicted backward mutations might be false-negative detections of mutations. In the case of inferred recurrent mutations, we found that the base assignments at these genomic positions were missing from many cells unaffected by the recurrent mutations. An elevated number of missing bases at these genomic positions and a high false-negative detection rate, potentially lead to the inference of recurrent mutations. Overall, while TopHap+ can detect recurrent mutations and losses of mutations and can be used for the analysis of mutational history, the inference needs to be carefully examined.

### 3.4 Analysis of CRISPR/Cas9 lineage tracing data using TopHap+

We lastly show that TopHap+ can also be used for data generated through CRISPR/Cas9-based gene editing with massively parallel single-cell readouts [28]. In this technology, genomic sites are designed to be targeted by Cas9 to induce indels, which are inherited by daughter cells. Thus, this technology is expected to be useful in tracking the movement of cells between tumor sites [28], [30].

**Fig. 7:**
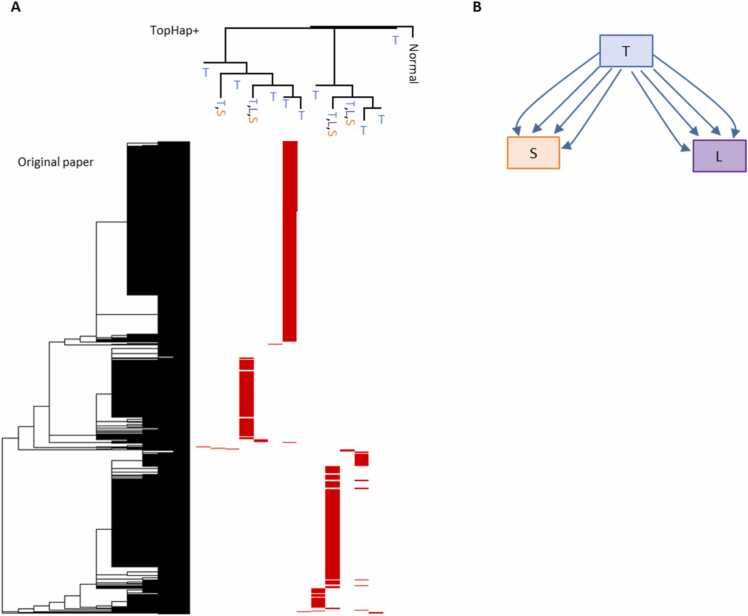
**Inferred clone phylogeny and metastatic cell migration events using CRISPR/Cas9-based gene editing data.** The 3724 dataset was used. (**A**) The TopHap+ clone phylogeny (top) was compared with the cell phylogeny inferred in the original study (left). The tips of the TopHap+ phylogenies indicate the tumor sites of the cells annotated to the clones. “T,” “S,” and “L” represent the primary tumor (lung), soft tissue, and lymph node, respectively. (**B**) PathFinder was used to infer metastatic cell migration history.

First, we describe the results of the 3724 dataset, where cells were sampled from the primary tumor (lung) and two metastatic tumors (liver and soft tissue) [28]. TopHap+ produced 13 clones, and the inferred clone phylogeny agreed well with the cell phylogeny reported in the original study [28], validating the performance of TopHap+ on CRISPR/Cas9-based lineage tracing data (Fig. 7**A**). In the TopHap+ phylogeny, all predicted clones were found within the primary tumor, and a few clones were found within the metastatic tumors in addition to the primary tumor. This observed pattern suggested that all clones likely evolved within the primary tumors, and some primary clones seeded the metastatic tumors. Indeed, the application of PathFinder predicted that both of the metastatic tumors were seeded from the primary tumor (Fig. 7**B**). Also, PathFinder predicted that there were multiple seeding events, i.e., four clones seeded for each of the metastatic tumors.

Next, we analyzed the three other datasets (3508, 3545, and 3515) and similarly found that TopHap+ phylogeny agreed well with the original study (Supplementary Fig. S6). Similar to the 3724 dataset, the primary tumors contained all or almost all clones identified, and PathFinder predicted that all metastatic tumors were seeded from the primary tumors with multi-seeding events. Overall, these results indicate that TopHap+ and PathFinder analysis can be also performed for CRISPR/Cas9-based gene editing data to reveal the dynamics of clones.

## 4 Discussion

In this study, we advanced the TopHap approach to analyze single-cell sequencing data. TopHap was originally developed to analyze SARS-CoV-2, where the data has only a small number of phylogenetically informative sites with > 1,000,000 sequences [19]. In such a data structure, phylogenetic signals compete with sequencing errors, which is the same as the issues observed in single-cell sequencing data analysis. The major advancement in the TopHap+ is the addition of mutation ordering analysis [21], to assess the reliability of the inference and resolve the evolutionary history of low-frequency mutations.

Analysis of empirical data using TopHap+ and PathFinder suggested that all tumor evolutions inferred in this study were affected by multi-clone seeding events. Currently, metastatic cell migration patterns are analyzed using tumor bulk sequencing data [7], [31], [32]. Although multi-clone seeding events have been reported previously, the number of such events was much smaller in the bulk sequencing data analysis [7]. Since bulk sequencing data analysis requires the decomposition of clone sequences, similar clones are difficult to be identified, which may result in an underestimation of the number of cell migration events. Overall, single-cell data can provide clone composition of tumors with higher resolution, and analysis of TopHap+ clone phylogeny can identify a more comprehensive cell migration history between tumor sites.

In the TopHap+ approach, we implemented mutation ordering analysis to test the reliability of inferred mutation order. Actually, our mutation ordering analysis can be coupled with other methods that infer mutation trees, e.g., SCITE. As an example, we coupled it with SCITE. We found that our mutation ordering analysis can also distinguish spuriously inferred mutation orders and can improve the inferences of mutation trees inferred by SCITE (**Supplementary Note** and Supplementary Fig. S7). Specifically, spurious clones were successfully filtered, and the clone count estimate became accurate (Supplementary Fig. S7A). Also, the accuracy of inferred clone phylogenies became comparable to TopHap+ (Supplementary Fig. S7B and S7C). We also tried to improve the CellPhy inference using the same mutation ordering analysis. However, this technique is based on inferred mutation orders unaffected by reversing and recurrent mutations. For the CellPhy trees, we were not able to use it because CellPhy incorrectly predicted that most of the mutations (>75%) were affected by reversing or recurrent mutations. Therefore, our mutation ordering analysis can be coupled with methods that are designed to order mutations. Since methods to assess the reliability of inferred mutation trees are currently lacking, our mutation ordering analysis will also be useful for the other methods to place reliability scores.

Lastly, a limitation of TopHap+ is the requirement of user-specified VAF and HF thresholds. While the default VAF and HF thresholds were set to 5 cells, more stringent thresholds must be assigned for a given dataset. For example, when more than half of inferred clones were removed through the mutation ordering analysis due to spurious mutation placements, TopHap+ analysis should be performed using higher VAF and HF thresholds (e.g., Supplementary Fig. S3). Also, to avoid a long computation time, more stringent thresholds may be desired when the number of low-frequency variants is very large (e.g., the analysis of the CRISPR/Cas9-based gene editing data). In the future, we plan to develop a method to determine an optimal VAF and HF for a given dataset. Also, detecting recurrent and reversing mutations from single-cell sequencing data is challenging due to a large number of missing bases and false-negative detections of mutations. Although TopHap+ can detect recurrent and reversing mutations, caution is necessary for these inferences.

In conclusion, TopHap+ employs the phylogenetic optimality principle and mutation ordering analysis to infer clone phylogeny, and it performs well. Also, our mutation ordering analysis can be employed to assess mutation trees inferred by other methods as well. Overall, the TopHap+ approach will be useful for revealing the mutational history, evolutionary relationship of cancer cell populations, and tumor biogeography.

## Funding

This research was supported by the 10.13039/100000002National Institutes of Health to S.K (LM-013385) and S.M (LM-014005). Publication of this article was funded in part by the Temple University Libraries Open Access Publishing Fund.

## Author contributions

S.M. and S.K. developed the method and designed research; T.D., S.M., and M.S. refined and implemented the technique; T.D. and S.M. performed analyses; and S.M., S.K., and T.D. wrote the article.

## Author statement

None.

## Declaration of Competing Interest

None.

## Data Availability

TopHap+ is available at https://github.com/SayakaMiura/TopHapPlus and the mutation ordering analysis to assess inferred mutation tree can be found at https://github.com/SayakaMiura/SCAN.
