# Supplementary material for: Improving cellular phylogenies through the integrated use of mutation order and optimality principles
Source: Comput Struct Biotechnol J. 2023 Aug 2;21:3894–903. doi: 10.1016/j.csbj.2023.07.018 (PMC10432911; doi:10.1016/j.csbj.2023.07.018)
Supplement: Supplementary file 1 — Supplementary material [file mmc1.pdf]

Supplementary Note

Mutation ordering analysis in TopHap+ approach

After the step of TopHap, a converted mutation tree may contain recurrent mutations, which are mapped to more than one branch, and reversing mutations (losses of mutations). For inferred reversing mutations, TopHap+ similarly computes COI with its direct ancestor mutation (e.g., mutation 3 for reversing mutation 0 in **Fig. S1**) and uses the same COI threshold as the other mutations to assess the reliability. In addition, TopHap+ retains reversing mutations only when COIs with all ancestral mutations (mutations 1 and 3) are significantly higher than those with sister mutations (mutations 2 and 4) using *t*-test because sister mutations should not co-occur with the reversing mutation.

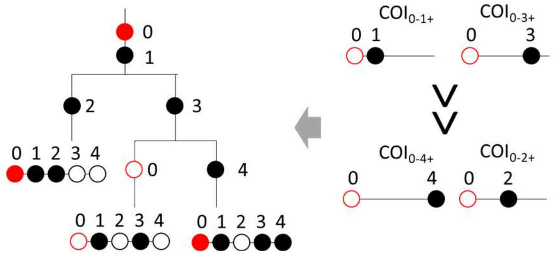

Figure S1. Example of loss of mutation. The red circle (mutation 0) represents the mutation that is affected by the loss of mutation. Filled and unfilled circles indicate the presence and absence of mutations, respectively.

For inferred recurrent mutations, TopHap+ requires that all COIs with their ancestor mutations (e.g., mutations 3 and 4 for recurrent mutation 0 in **Fig. S2**) should be higher than the desired threshold (e.g., 0.1). Only when these COIs are significantly higher than a COI threshold by *t*-test ($p < 0.05$), the recurrent mutation is retained.

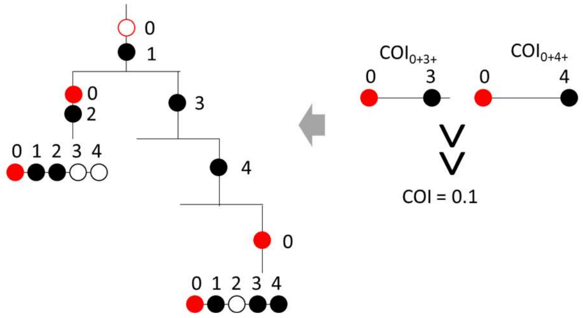

Figure. S2. Example of recurrent mutation. The red circle (mutation 0) represents the mutation that is affected by recurrent mutation. Filled and unfilled circles indicate the presence and absence of mutations, respectively.

At the step of attaching excluded mutations using the mutation ordering analysis, inferred recurrent and reversing mutations are temporarily removed, because their COIs are affected. These temporarily removed mutations are added back to the same positions in the mutation tree after attaching all excluded mutations. Thus, TopHap+ does not identify any additional recurrent and reversing mutations at the step of attaching excluded mutations. Also, attached mutations that create branches with single mutations are removed, because those attachments are often erroneous in the mutation ordering analysis. Last, TopHap+ calculates COI for all pairs of

mutations in a dataset. When a mutation that is predicted to be a descendant (*b*) always has high COIs with the other mutations (>0.6 was used in this study), it indicates that the mutation is found in most of the cell sequences. Thus, such a mutation should have occurred at the earliest time and these mutations are assigned to the trunk of the mutation tree.

Mutation ordering analysis coupled with other methods (SCAN approach)

SCAN is designed to assess the reliability of an inferred mutation tree from an available method. The input mutation tree is required to have all cells attached to nodes (mutations) of a given mutation tree. Using the same strategy as TopHap+, SCAN tests if each predicted ancestor-descendant (sequential) relationship is reliable using the observed and expected number of cells with the early mutation without the later mutation (C_{a-} and E_{a-}, respectively) and the observed and expected number of cells that carry both earlier and later mutations (C_{ad} and E_{ad}, respectively) (**Fig. 2H-2J**). A given mutation tree is accordingly refined. Also, SCAN computes COI for each pair of mutations and prunes mutations with the desired COI threshold (**Fig. 2D-2F**). A refined mutation tree is lastly converted to a clone phylogeny. Cells are excluded when they are attached to mutations that are pruned or that are predicted to be intermediate. Also, SCAN excludes cells that contain a larger number of sequencing errors than other cells, i.e., potentially doublets. Using the same approach in TopHap+, SCAN estimates the FPR_i and FNR_i for each cell sequence, *i*. When either the FPR_i or FNR_i of a cell is significantly higher than the other cells ($p < 0.05$ by *t*-test), the cell is annotated as spurious.

Supplementary Figures

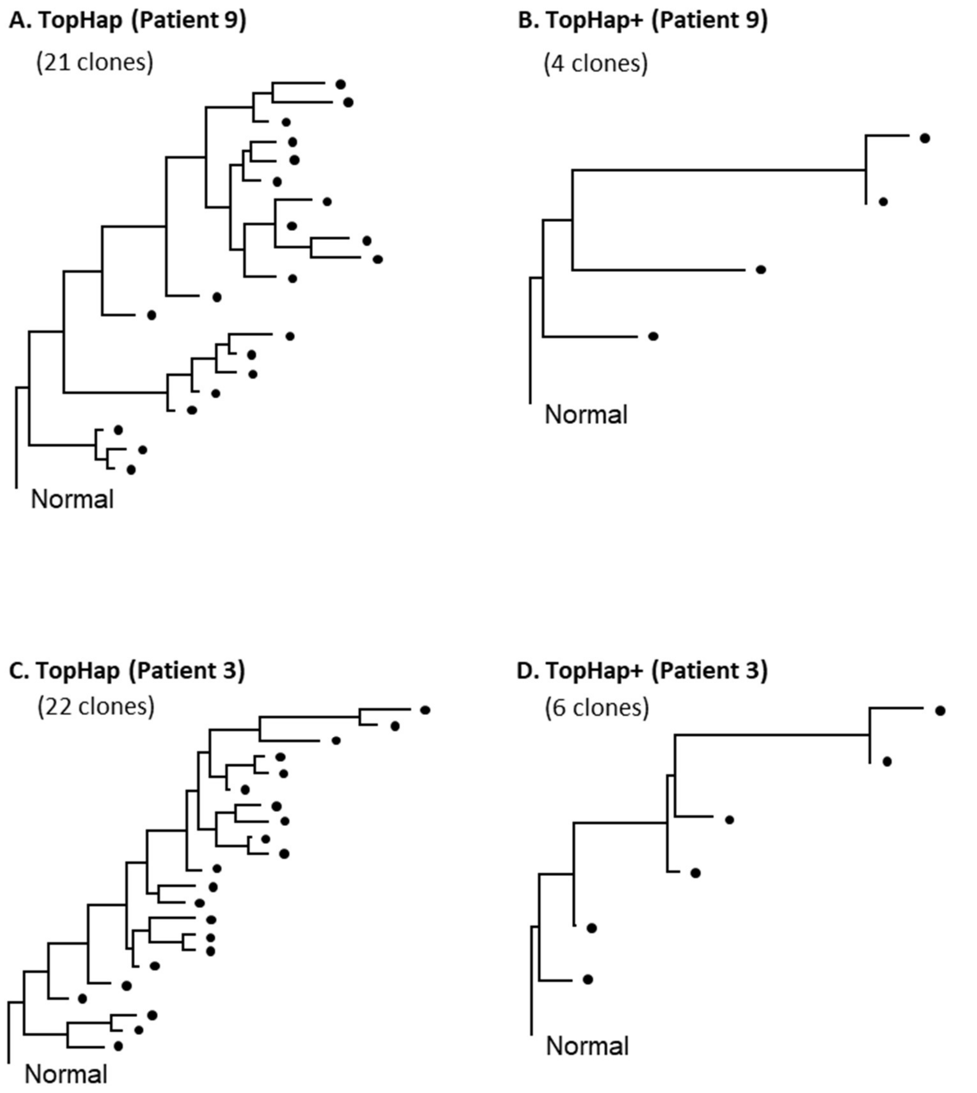

Figure. S3. TopHap+ analysis with VAF and HF thresholds of 5 cells for Patient 3 and 9 datasets. The Patient 9 (**A** and **B**) and Patient 3 datasets (**C** and **D**) were used. (**A** and **C**) TopHap phylogenies. (**B** and **D**) TopHap+ phylogenies. After the COI filtering, more than half of the clones were removed, indicating that the initial TopHap phylogeny is potentially erroneous. We recommend to increase the VAF and HF threshold in the TopHap+ analysis. Circles are clones.

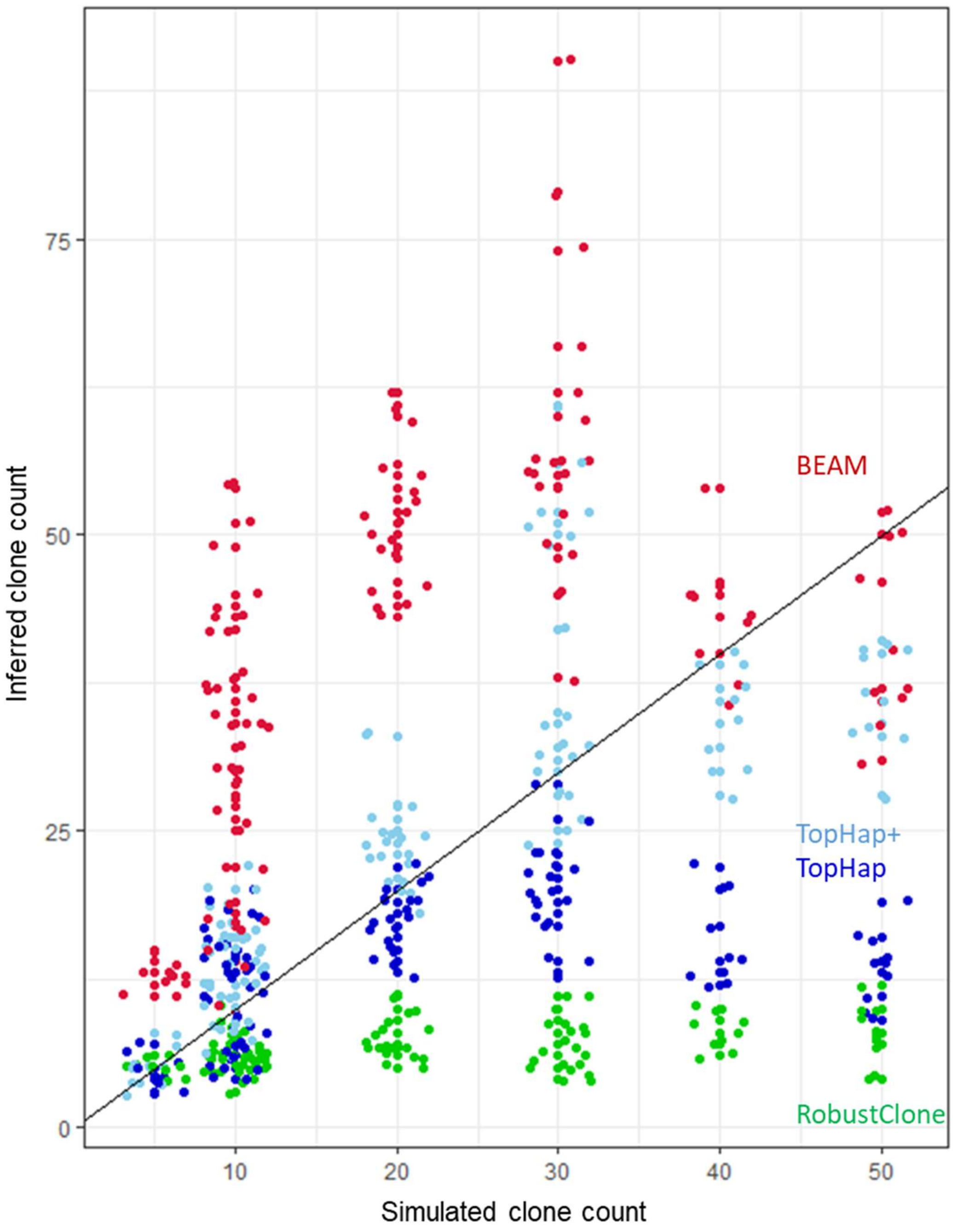

Figure. S4. Simulated and inferred clone counts. Each dot represents a dataset. The black line is the one-to-one line.

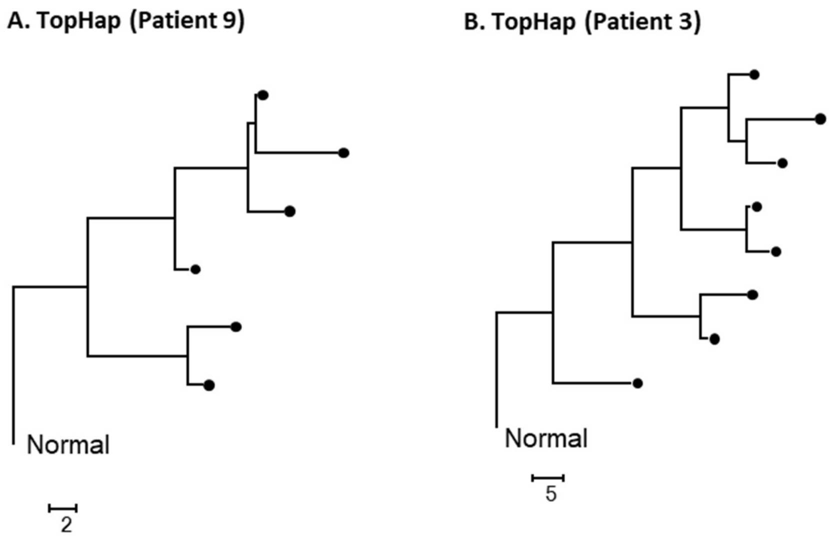

Figure. S5. TopHap analysis with 5% VAF and 10 cells HF thresholds for Patient 3 (A) and 9 datasets (B). Circles are clones.

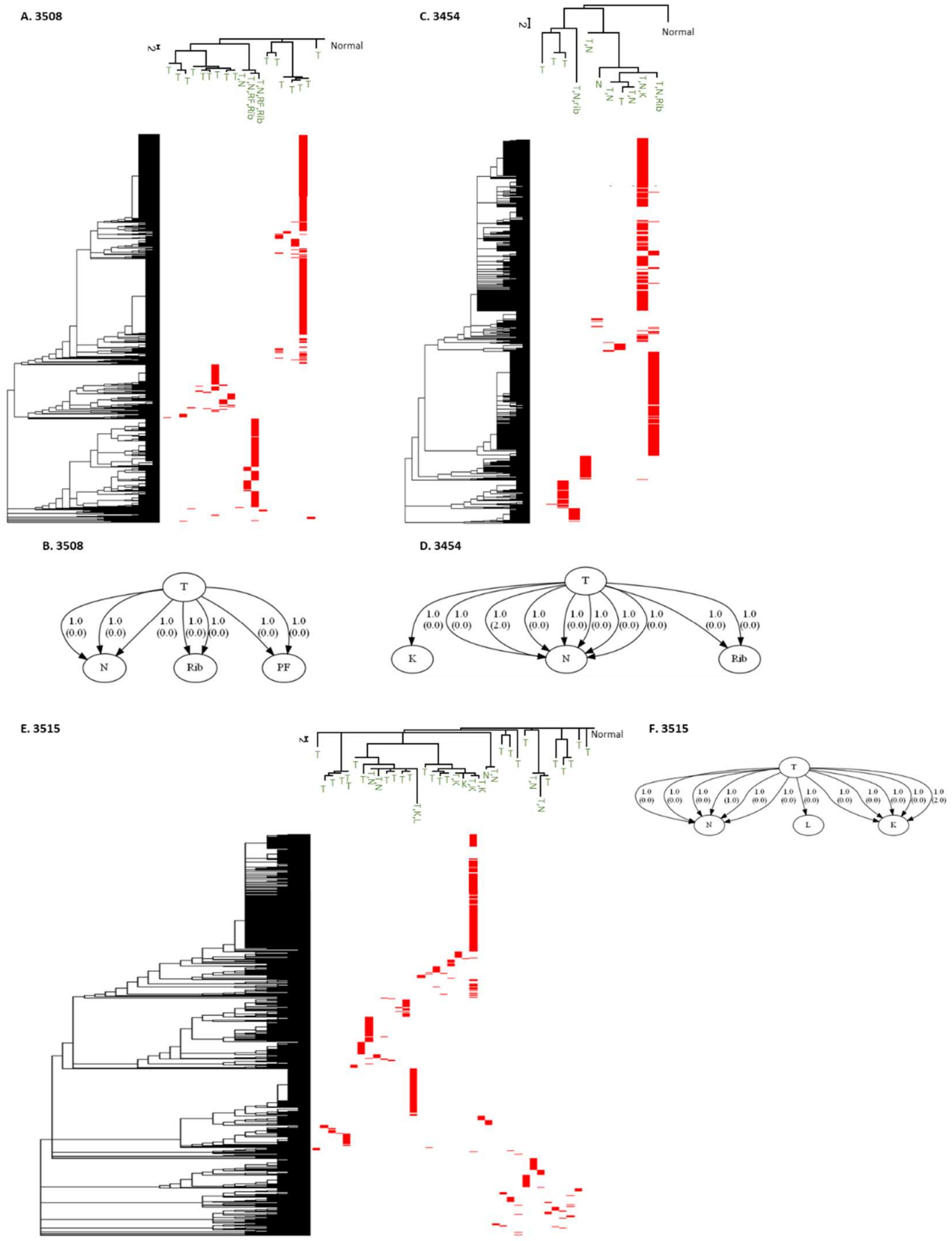

Figure S6. Inferred clone phylogeny and metastatic cell migration events using 3508 (A and B), 3454 (C and D), and 3515 (E and F) datasets generated through CRISPR/Cas9-based gene editing. (**A**, **C**, and **E**) The TopHap+ phylogenies (top) were compared with those inferred in the original study (left). The tips of the TopHap+ phylogenies indicate the tumor sites of the cells that were annotated to the clones. “T,” “S,” “L,” “N,” and “K” represent the primary tumor (lung), soft tissue, liver, lymph node, and kidney, respectively. (**B**, **D**, and **F**) PathFinder was used to infer metastatic cell migration history. A number next to an arrow is a PathFinder’s reliability score, and the number of mutations that are associated with a cell migration event is shown within parenthesis.

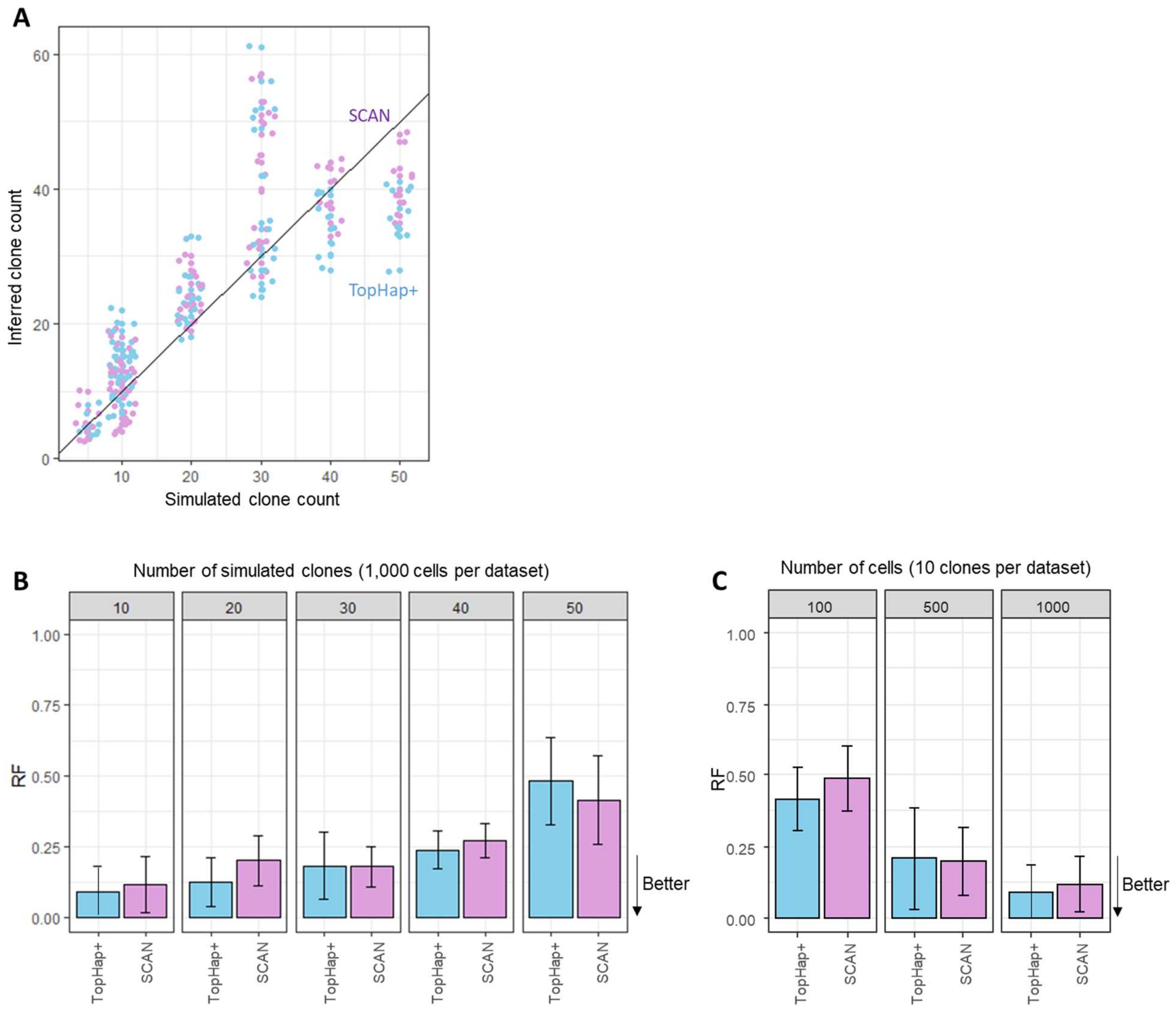

Figure S7. The performance of our mutation ordering analysis when coupled with SCITE (SCAN). (**A**) Simulated and inferred clone count per dataset. Each point represents each dataset. The black line is the one-to-one line. (**B** and **C**) Accuracy of inferred clone phylogeny between different numbers of simulated clones (**B**) and between different numbers of cells in dataset (**C**). RF was computed between inferred and simulated clone phylogenies for each method. The average RF across the datasets is plotted, and an error bar represents the standard deviation. We included only datasets with at least three inferred clones. For SCAN, the COI of 0.4 threshold was used.
